# Amelioration of Cadmium-Produced Teratogenicity and Genotoxicity in Mice Given *Arthrospira maxima* (Spirulina) Treatment

**DOI:** 10.1155/2013/604535

**Published:** 2013-11-28

**Authors:** Nancy Argüelles-Velázquez, Isela Alvarez-González, Eduardo Madrigal-Bujaidar, Germán Chamorro-Cevallos

**Affiliations:** ^1^Laboratorio de Toxicología Preclínica, Departamento de Farmacia, Escuela Nacional de Ciencias Biológicas, Instituto Politécnico Nacional, Avenida Wilfrido Massieu s/n, Unidad Adolfo López Mateos, Delegasión Gustavo A. Madero, 07738 México, DF, Mexico; ^2^Laboratorio de Genética, Departamento de Morfología, Escuela Nacional de Ciencias Biológicas, Instituto Politécnico Nacional, Avenida Wilfrido Massieu s/n, Unidad Adolfo López Mateos, Delegasión Gustavo A. Madero, 07738 México, DF, Mexico

## Abstract

Evaluation of the effects of *Arthrospira maxima* (AM) was made, otherwise known as Spirulina, on the teratogenicity, genotoxicity, and DNA oxidation processes induced by cadmium (Cd). Pregnant ICR mice were divided into groups and administered water, Cd only, AM only, or AM plus Cd. AM was administered orally at doses of 200, 400, and 800 mg/kg from gestational day 0 (GD0) to GD17, and at GD7 there was an intraperitoneal challenge of Cd (1.5 mg/kg). Cd only caused fetal malformations, including exencephaly, micrognathia, ablephary, microphthalmia, and clubfoot, as well as a significant increase in the quantity of micronucleated polychromatic erythrocytes (MNPE) and of micronucleated normochromatic erythrocytes (MNNE) in blood cells of both the mothers and their fetuses. An increased level of oxidation was also found, measured by a rise in the levels of the adduct 8-hydroxy-2-deoxyguanosine. In a dose-dependent manner, AM significantly reduced the number of external, visceral, and skeletal malformations, the quantity of MNPE and MNNE, and the level of DNA oxidation. The results suggest that AM may reduce the genotoxic effects and rates of congenital malformations caused by exposure to Cd *in utero* and that the antioxidant activity of this cyanobacterium could be responsible, at least in part, for producing this effect.

## 1. Introduction

Nowadays, widespread environmental exposure to cadmium (Cd) represents a public health problem. This metal, which is derived from both natural and anthropogenic sources, is capable of causing adverse effects [1], such as cancer, metabolic and cardiovascular disorders, and reproductive defects.

Cd has been classified as a human carcinogen by the International Agency for Research on Cancer and the German MAK Commission [2]. Exposure to Cd significantly increased the frequency of micronuclei in polychromatic erythrocytes in both tibia bone marrow and peripheral blood in rats [3]. Genotoxicity was also induced by the exposure of zebra fish to Cd at environmentally relevant doses [4]. Regardless of the time of exposure, human contact with Cd significantly increased chromosomal aberrations and exchange of sister chromatids in all age groups [5].

Cd has been shown to target multiple organs and systems following intoxication, causing nephrotoxicity, immunotoxicity, and osteotoxicity [6]. It also leads to some diabetic complications and adverse cardiovascular effects [7, 8]. In animal models and humans, Cd has been shown to enter the brain parenchyma and neurons, which causes neurological alterations that result in a lower attention span, hypernociception, olfactory dysfunction, and memory deficits [9].

Finally, it is known that Cd induces embryotoxicity, including harmful effects on growth and mortality, as well as a wide range of congenital malformations in laboratory animals [10–12]. Malformations have also been found in animal species in nature, such as *in Xenopus *embryos [13]. Exposure during neurulation results in the formation of neural tube defects in all species, the extent of which depends on dose, time of exposure, strain, and nutritional status [12, 14–16]. Other effects include male and female subfertility or abortions [17, 18]. Moreover, Kippler et al. [19] reported that maternal exposure to Cd was significantly and inversely associated with the head circumference and birth weight of girls.

Some toxic effects of Cd are a result of its capacity to stimulate oxidative stress [20, 21] by interacting with the thiol groups of antioxidant enzymes (demonstrated *in vivo* and *in vitro*), and thus inhibiting the latter [2]. The administration during gestation and lactation of natural antioxidants, including vitamin E, carotenoids, vitamin B_6_, and zinc, has prevented a number of the negative effects of Cd [22]. The antioxidant effects of isoprenoids, found in various aromatic plants, royal jelly, and grapefruit juice, reportedly protect against Cd-induced genotoxicity and oxidative stress in mice [23–25]. Moreover, it has been shown in several studies that *Arthrospira maxima* (AM), commonly known as Spirulina, helps to counter the harmful effects of Cd through antioxidant effects in experimental animal models [26, 27].

AM is a microscopic and filamentous cyanobacteria within the Oscillatoriaceae algae family. It makes a positive contribution to human and animal nutrition, providing a rich source of proteins and vitamins, especially B12 and provitamin A, essential amino acids, minerals, essential fatty acids, glycolipids, sulfolipids, phycocyanin, eicosapentaenoic acid, and other phytochemicals [28, 29].

Several studies have demonstrated that AM possesses significant antidiabetic, immunomodulatory, anti-inflammatory, antiviral, anticancer, and antibacterial properties [30–33]. It has also proven to be an effective treatment for certain types of anemia, hepatotoxicity, cardiovascular diseases, and hyperlipidemia [34]. Additionally, it is possible that AM provides neuroprotection in patients with Parkinson's disease [35] and helps to preserve the functions of the central nervous system, including memory, depression, and motor activity, in individuals at an advanced age [36–38]. It has been demonstrated that the majority of these effects are due to antioxidant activity [39].

On the basis of these reports, the present study was undertaken to determine the possible protective effect of AM for ICR mice in a model of depression induced by Cd oxidative teratogenicity and genotoxicity.

## 2. Materials and Methods

### 2.1. Materials

Analytical grade Cd (specifically CdCl_2_), alcian blue, and alizarin red were purchased from Sigma-Aldrich Chemicals Co (St. Louis, MO, USA). Urea, ethanol, acetone, Tween 80, and potassium hydroxide were obtained from J.T. Baker Chemicals (Mexico City). Acetic acid, picric acid, Giemsa, and formaldehyde were purchased from Merck, S.A. de C.V (Mexico City). The HT 8-hydroxy-2-deoxyguanosine (8-oxo-dG) ELISA Kit was obtained from Trevigen, Inc. (Gaithersburg, MD, USA) for the detection and quantification of the 8-oxo-dG adduct in serum samples. The AM sample was obtained from a bulk production batch (5DW-9714) of standard quality, supplied by Alimentos para la Humanidad, S.A. de C.V (Mexico City).

### 2.2. Animals

Male and female ICR mice (Harlan de México, S.A. de C.V) weighing 30 ± 2 g were kept on a 12:12 h light/dark cycle (lights on at 9:00 am) at a constant temperature of 24 ± 1°C and with 50 ± 10% relative humidity. Animals were fed Purina Rodent Lab Chow (St. Louis, MO, USA) and provided with water ad libitum. The experimental study was approved by the Committee of Bioethics of the National School of Biological Sciences and conducted in compliance with the Mexican Official Standard (NOM ZOO-062-200-1999) regarding technical specifications for production, care, and use of laboratory animals.

### 2.3. Experimental Protocol

One male was housed with two nulliparous females from 6:00 to 9:00 am (the last 3 hours of the dark cycle). The presence of a vaginal plug was regarded as evidence of coitus, with the day of discovery designated as gestational day 0 (GD0). 

Pregnant females were randomly divided in 7 groups of 8 animals each, as shown in Table 1.

Cd was dissolved in water and administered to pregnant mice by intraperitoneal (ip) injection on GD7. The dose of 1.5 mg/kg dose was previously found to produce exencephaly in mouse fetuses [40]. AM suspended in 0.95% Tween 80 was given to mice daily by intragastric administration from GD0 to GD17. The doses selected had been very well tolerated by mice in previous studies [41]. In all cases, a constant volume of 1 mL/100 g of body weight was administered. Maternal weight was monitored on the following gestational days: 0, 7, 11, 15, and 17.

### 2.4. Teratogenic/Antiteratogenic Assessment

Pregnant mice were sacrificed by cervical dislocation on GD17 and the whole uterus was removed. The numbers of implantation sites, resorptions, and dead and live fetuses were recorded. Fetuses and placentas were weighed and measured, and the fetuses were inspected for external congenital malformations. Two-thirds of the offspring was subjected to visceral examination for the detection of other anomalies. These fetuses were first fixed in Bouin's solution, and then sections were prepared as described by Wilson [42]. The remaining fetuses were fixed in 95% alcohol for skeletal staining with alcian blue and alizarin red, which allowed for the differentiation between cartilage and ossified skeleton [43]. In either case, they were examined under a Zeiss dissection stereomicroscope.

One litter was used as the control to establish maternal parameters (implantations, resorptions, live fetuses, placental diameter, and placental weight). The *χ* ^2^-test followed by Fisher's exact test was employed to compare experimental groups to the control, evaluating abnormalities of fetuses as well as visceral and skeletal anomalies. The remaining variables were assessed by one-way ANOVA and then by Student Newman Keuls test (post hoc test). A value of *P* < 0.05 was considered significant.

### 2.5. Micronuclei/Antimicronuclei Assessment

A blood sample was taken from the tail of each of 8 pregnant mice and smeared on a clean slide. A neck incision was made in 4 fetuses per mother. From the blood collected in a capillary tube from each fetus, one drop was smeared on two clean slides. The remaining blood was centrifuged at 3000 rpm for 15 min to separate the serum, which was kept at −70°C to await the determination of DNA oxidative damage.

The slides with blood samples from mothers and fetuses were fixed in methanol for 3 min and then washed, dried, and stained for 12 min with 10% Giemsa solution made with PBS. To evaluate the genotoxic effect induced by Cd, as well as the protection provided by AM, 1000 polychromatic erythrocytes from in each organism were analyzed and the number of micronucleated cells (MNPE) was recorded. Likewise, 1000 normochromatic erythrocytes from each organism were analyzed and the frequency of micronucleated cells (MNNE) was recorded. Statistical analysis of the results was made with ANOVA followed by the Tukey-Kramer test.

### 2.6. Quantification of 8-oxo-dG

The *quantification of 8-Oxo-dG* was carried out in fetal serum from mice treated as indicated in Section 2.3, following the instructions of the HT 8-oxo-dG ELISA Kit. The calibration curve and the effect of the test compounds were determined in duplicate.

In this immunoassay, the 8-oxo-dG monoclonal antibody binds competitively to 8-oxo-dG immobilized on precoated wells and in solution. The antibody bound to 8-oxo-dG in the sample is washed away, while the antibody bound to 8-oxo-dG attached to the well is retained. Detection is performed with the HRP conjugate and tetramethylbenzidine substrate. Absorbance was measured in a microplate reader at 450 nm, with the intensity of yellow being inversely proportional to the concentration of 8-oxo-dG. The standard curve was used for calculating the average concentration of 8-oxo-dG for each sample. For this calculation, we used the worksheet at http://www.trevigen.com/8-oxo-dG/ELISA.php.

## 3. Results

### 3.1. Teratogenic/Antiteratogenic Study

Results of maternal toxicity are shown in Table 2. Cd intoxication (applied on GD7) produced a significant decrease (*P* < 0.05) in the weight of the pups and in fetal crown-rump length. However, none of the other variables, such as maternal weight, implantations, and resorptions per mother, were altered in comparison with the control group. Administration of AM alone did not induce any of these alterations.

The incidence and type of each of the external malformations are shown in Table 3. The malformations produced by Cd included exencephaly, micrognathia, ablephary, microphthalmia, and clubfoot. AM treatment (at 200, 400, and 800 mg/kg) significantly decreased the frequency of these malformations, reducing them to zero in the cases of ablephary, microphthalmia, and clubfoot.

Visceral examination showed that Cd induced head and kidney abnormalities, such as hydrocephaly and head hypoplasia (Table 4). AM significantly reduced the frequency of these abnormalities compared to the group treated only with Cd.

Regarding skeletal anomalies, the most striking effects were seen in the offspring of the Cd only group, including delayed ossification of the head and vertebrae, as well as fused ribs and vertebrae (Table 5). AM treatment at all doses significantly diminished (*P* < 0.05) the number of fetuses with external, visceral, and skeletal abnormalities in a dose-dependent manner.

### 3.2. Micronuclei/Antimicronuclei Study

Results of the micronucleus assay in pregnant mice and their fetuses are presented in Table 6. A mean of 2.16 MNPE was found in adult mice administered AM only, a value similar to that observed in the control group. On the contrary, animals administered Cd only showed a 7-fold increase in this value. Mice treated with AM plus Cd exhibited a dose-dependent reduction in the number of MNPE. These reduced levels of MNPE represented a statistically significant difference compared with the level found in animals treated with Cd only. The best protection (65.9%) was observed with the highest test dose (800 mg/kg) of AM.

The evaluation of MNNE levels showed a pattern similar to that described previously, although with higher values. This result is logical, considering that MN registered in normochromatic erythrocytes include those which have accumulated over time. The administration of Cd only increased the MNNE level more than four times that found in the control, and the administration of AM plus Cd produced a dose-dependent decrease in the number of MN observed in the group treated with Cd only. The best protection (40.3%) was obtained with the highest test dose of AM. With regard to the fetuses, we found a mean of 4.69 and 29.7 for MNPE and MNNE, respectively, for the control and AM only groups. With the administration of Cd to mice, there was a greater than three- and fourfold increase in MNPE and MNNE levels, respectively. With the administration of AM plus Cd, a dose-dependent decrease was determined in both types of erythrocytes compared to the group treated with Cd only.

### 3.3. Quantification of 8-Oxo-dG

**Figure 1:**
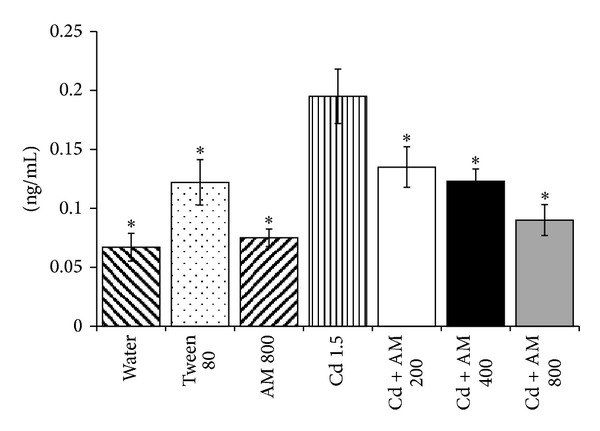
Effect of *Arthrospira maxima *(AM) on the level of 8-hydroxy-2-deoxyguanosine (8-oxo-dG) induced by Cd in the serum of mice fetuses. 8-oxo-dG was measured in fetal serum samples by means of HT 8-oxo-dG ELISA Kit. Bars represent means ± SEM of 5 groups with six individuals each. The calibration curve, as well as the effect of the tested compounds, was determined by duplicate. For the quantification, we followed the instructions shown the HT 8-oxo-dG ELISA Kit. *Statistically significant difference with respect to the value of Cd treated mice. One-way ANOVA and Student Newman Keuls tests; *P* < 0.05.

Figure 1 shows the mean concentration of 8-oxo-dG detected in the serum of fetuses. Mice treated with water had a low value of DNA oxidation (0.06 ng/mL), and a similar value was observed in animals administered 800 mg/kg of AM. However, animals administered Tween 80 showed about a twofold increase in the value for DNA oxidation.On the other hand, in Cd treated mice there was a 40% increase with respect to the level found in the Tween 80 control and a 120% increase with respect to the level in mice treated with water. On the other hand, mice administered AM (at doses of 200, 400, and 800 mg/kg) plus Cd showed a dose-dependent decrease with respect to the group treated with Cd only. This decrease was 130% with the highest test dose of AM, representing the best protective effect.

## 4. Discussion

We evaluated the teratogenic and genotoxic effects of Cd in pregnant mice and their embryos, as well as the potential protection provided by AM treatment. The intraperitoneal administration of Cd to ICR mice (at a dose of 1.5 mg/kg on GD7) produced exencephaly, micrognathia, ablephary, microphthalmia, and clubfoot in the offspring, indicating embryonic sensitivity to low levels of the teratogenic agent. Contrarily, there was no increase in the aforementioned parameters with the administration of AM alone (800 mg/kg) compared to the control group or AM (200, 400, and 800 mg/kg) plus Cd compared to the group treated with Cd only. These results demonstrate that teratogenicity owed itself only to the direct effect of Cd (and not of AM) on embryonic development and thus coincide with the exencephaly found in a previous study, in which Cd exposure before neurulation caused an opening in the anterior neural pore [1]. However, the frequency of this alteration has been variable in different studies due to distinct experimental conditions.

The mechanism by which the exposure of a mother to Cd could affect the weight of pups remains controversial. Several hypotheses have been proposed, such as interference with the transport of essential minerals, placental damage, endocrine disruption, interaction with essential elements, or a combination of these factors [44, 45].

It is known that the teratogenicity of Cd is influenced by the simultaneous administration of other chemical agents or the previous administration of the same. For example, it has been reported that pretreatment with Cd protects animals to some extent from the teratogenicity of a subsequent administration of a higher dose of the same metal [46]. Moreover, simultaneous administration of Cd with metallothionein or glycine has shown protection against the harmful effects of the former [26, 47].

In the current study, the coadministration of AM (at all doses) significantly reduced the frequency of external abnormalities produced by the administration of 1.5 mg/kg of Cd. With the highest dose of AM, some parameters indicating the effect of Cd reached values similar to those of the control group. The cases of exencephaly and micrognathia are quite notable, as their frequency with the AM plus Cd treatment was 3.3 and 1.1%, respectively, compared with 60.8 and 51.0%, respectively, in the groups treated with Cd only. This result together with the dose-dependent effect strongly suggests that the alga has a high potency for protection against damage caused by Cd.

Although the number of Cd-produced malformations was not high, AM treatment at all doses significantly diminished these negative effects. The same was observed with skeletal anomalies, consisting mainly of delayed ossification. Regarding the possible negative effect of the administration of AM alone, no alteration was found in any of the fetuses of this group. This coincides with our previous results on safety conducted on different species of rodents, in which AM constituted up to 30% of the animals' diet [48, 49].

With regard to MN, our present results on pregnant mice and their products agree with the strong clastogenic potential of Cd shown in various *in vitro* and *in vivo* models. For example, a previous study on mouse fetuses demonstrated the transplacental genotoxic effect of Cd (thus extending knowledge of this effect, which was previously reported with regard to benzene, vinblastine, benzo(a)pyrene, and verapamil) [50, 51]. Other reports also emphasize the importance of evaluating the transplacental genotoxic capacity of substances because numerous agents may cross the placenta and damage the fetus, such as antineoplastics, anticonvulsivantes, virus, hormones, altered maternal nutrients, or malignant cells [52, 53]. The present results demonstrate the relevance of finding agents that avoid or reduce the damage of the fetus, as well as the relevance of determining adequate doses for chemopreventive or therapeutic agents in pregnancy.

The intracellular production of ROS has been considered one of the main causes of cadmium toxicity. This effect has been demonstrated in a number of organs, including liver, kidney, lung, testis, and brain [9], through the induction of lipid peroxidation, protein carbonylation, mitochondrial alterations, a decrease in endogenous and exogenous antioxidants, and an increase in DNA oxidation [20]. These reports underline the urgency of finding antioxidant agents for preventing or reducing cadmium toxicity.

With regard to this strategy, it is interesting to note the positive effect observed with vitamin C or quercetin [54], constituents of grapefruit that have antigenotoxic properties. Recently, attention has been paid to the antioxidant potential of AM. This mechanism of action has shown pronounced effects on cadmium-intoxicated rats, reducing oxidative stress parameters such as malondialdehyde, conjugated diene, and hydroperoxide [55]. AM has proved to increase the level of antioxidant enzymes, including superoxide dismutase, glutathione peroxidase, and reduced glutathione [56, 57]. Indeed, antioxidant properties have been demonstrated for many of the chemical components of this cyanobacterium, such as phenolic compounds, tocopherols, *β*-carotenes, *γ*-linolenic acid, and phycocyanin [34, 39, 58].

In particular, phycocyanin has been found in a high concentration and has shown a powerful antioxidant activity in different studies [57, 59, 60]. This is congruent with the sharp reduction in the amount of 8-oxo-dG induced by AM treatment in the present study, considering that this adduct is a sensitive biomarker for DNA oxidative damage. Such damage adversely affects the DNA function via anomalies in gene transcription or mutations [61]. This action is relevant to the current results because Cd is known to increase the number of 8-oxo-dG adducts in adult and fetal mammalian cells, and such an increase has been related, at least in part, to observed genotoxic and teratogenic damages [25, 62, 63].

Based on this information, we propose that the protective effects observed in the present study can be attributed, at least partially, to the action of the antioxidant constituents of AM. The current results suggest that under conditions of low dose exposure of Cd, dietary supplementation with this cyanobacterium during gestation may help prevent complications associated with teratogenesis and mutagenesis. However, further study is needed before these results can be extrapolated to humans.

## Figures and Tables

**Table 1:** 

Groups	Treatment (mg/kg of body weight)
I	Water
II	0.95% Tween 80
III	Cd (1.5) only
IV	AM (800) only
V	AM (200) + Cd (1.5)
VI	AM (400) + Cd (1.5)
VII	AM (800) + Cd (1.5)

**Table 2:** Effects of treatment with cadmium (Cd) and *Arthrospira maxima* (AM) on pregnancy and reproductive outcome in mice.

Treatment (mg/kg)	Maternal weight gain (g)	Implantations per mother	Resorptions per litter	Live fetuses	Fetal crown-rump length (cm)	Fetal weight (g)	Placental diameter (cm)	Placental weight (mg)
Water	28.12 ± 1.29	13.11 ± 0.5	0.53 ± 0.13	96	2.09 ± 0.07	1.21 ± 0.42	0.81 ± 0.03	0.10 ± 0.01
Tween	29.11 ± 1.30	13.21 ± 0.7	1.02 ± 0.67	95	2.14 ± 0.08	1.23 ± 0.20	0.80 ± 0.03	0.10 ± 0.02
AM (800)	29.62 ± 1.12	12.67 ± 0.5	0.97 ± 0.14	94	2.12 ± 0.11	1.16 ± 0.31	0.81 ± 0.02	0.10 ± 0.01
Cd (1.5)	25.12 ± 1.93	13.33 ± 0.4	1.86 ± 1.23	92	1.90 ± 0.06^a^	0.97±0.22^a^	0.79 ± 0.07	0.09 ± 0.02
AM + Cd (200)	27.32 ± 1.52	13.03 ± 0.9	1.79 ± 0.78	98	1.98 ± 0.02^a^	1.01 ± 0.36^a^	0.80 ± 0.01	0.10 ± 0.01
AM + Cd (400)	27.45 ± 1.35	12.72 ± 0.6	1.54 ± 0.84	93	1.97 ± 0.01^a^	1.11 ± 0.31^a^	0.81 ± 0.02	0.09 ± 0.01
AM + Cd (800)	30.04 ± 0.78	12.65 ± 0.3	1.40 ± 0.77	90	2.02 ± 0.02	1.13 ± 0.41	0.78 ± 0.03	0.10 ± 0.03

The determinations were made with 8 pregnant mice per group. Results are shown as the mean ± SEM. Values inside parenthesis represent percentage. ^a^Statistically significant difference with respect tothe control value and ^b^with respecttothe value of Cd treated mice. One-way ANOVA and Student Newman Keuls tests; *P* < 0.05.

**Table 3:** Incidence and type of malformations at term induced by cadmium (Cd) and *Arthrospira maxima* (AM) in mice.

Treatment (mg/kg)	Examined fetuses	Number of fetuses with external abnormalities				
		Exencephaly	Micrognathia	Ablephary	Microphthalmia	Clubfoot
Water	96	0^b^	0^b^	0^b^	0^b^	0^b^
Tween	95	0^b^	0^b^	0^b^	0^b^	0^b^
AM (800)	94	0^b^	0^b^	0^b^	0^b^	0^b^
Cd (1.5)	92	56 (60.86)^a^	47 (51.08)^a^	11 (12.50)^a^	8 (8.69)^a^	26 (28.26)^a^
AM + Cd (200)	98	13 (13.26)^a,b^	2 (2.04)^a,b^	1 (1.020)^a,b^	3 (3.06)^a,b^	3 (3.06)^a,b^
AM + Cd (400)	93	6 (6.45)^a,b^	1 (1.07)^a,b^	0^b^	1 (1.098)^a,b^	1 (1.098)^a,b^
AM + Cd (800)	90	3 (3.33)^a,b^	1 (1.11)^a,b^	0^b^	0^b^	0^b^

The determinations were made with 8 pregnant mice per group. Values inside parenthesis represent percentage. ^a^Statistically significant difference with respect to the control value and ^b^with respect to the value of Cd treated mice. *X* ^2^-test followed by Fisher's exact test; *P* < 0.05.

**Table 4:** Effect of maternal exposure to cadmium (Cd) and *Arthrospira maxima* (AM) on the visceral morphology of fetus.

Treatment (mg/kg)	Examined fetuses	Number of fetuses with visceral abnormalities			
		Head		Kidney	
		Hydrocephaly	Hypoplasia	Ectopic	Hypoplasia
Water	63	0^b^	0^b^	0^b^	0^b^
Tween	64	0^b^	0^b^	0^b^	0^b^
AM (800)	62	0^b^	0^b^	0^b^	0^b^
Cd (1.5)	63	5 (7.93)^a^	5 (7.93)^a^	9 (14.28)^a^	6 (9.52)^a^
AM + Cd (200)	61	3 (4.91)^a,b^	2 (3.27)^a,b^	7 (11.47)^a,b^	4 (6.55)^a,b^
AM + Cd (400)	64	2 (3.12)^a,b^	1 (1.56)^a,b^	4 (6.25)^a,b^	3 (4.68)^a,b^
AM + Cd (800)	63	0^b^	0^b^	1 (1.58)^a,b^	1 (1.58)^a,b^

The determinations were made with 8 pregnant mice per group. Values inside parenthesis represent percentage. ^a^Statistically significant difference with respect to the control value and ^b^with respect to the value of Cd treated mice. *X* ^2^-test followed by Fisher's exact test; *P* < 0.05.

**Table 5:** Effects of maternal exposure to cadmium (Cd) and *Arthrospira  maxima* (AM) on fetal skeletal morphology of mice.

Treatment (mg/kg)	Examined fetuses	Number of fetuses with delayed ossification				Fused	
		Head	Vertebrae				
			Sternebrae	Cervical	Sacra	Ribs	Vertebrae
Water	33	0^b^	0^b^	0^b^	0^b^	0^b^	0^b^
Tween	32	0^b^	0^b^	0^b^	0^b^	0^b^	0^b^
AM (800)	32	0^b^	0^b^	0^b^	0^b^	0^b^	0^b^
Cd (1.5)	30	16 (73.33)^a^	9 (30)^a^	7 (23.33)^a^	8 (26.66)^a^	7 (23.33)^a^	13 (43.33)^a^
AM + Cd (200)	31	5 (25.80)^a,b^	6 (19.35)^a,b^	6 (19.35)^a,b^	5 (16.12)^a,b^	6 (19.35)^a,b^	9 (29.03)^a,b^
AM + Cd (400)	31	2 (9.67)^a,b^	4 (12.90)^a,b^	6 (19.35)^a,b^	5 (16.12)^a,b^	4 (12.90)^a,b^	7 (22.58)^a,b^
AM + Cd (800)	30	0^b^	2 (6.66)^a,b^	3 (10)^a,b^	2 (6.66)^a,b^	2 (6.66)^a,b^	2 (6.66)^a,b^

The determinations were made with 8 pregnant mice per group. Values inside parenthesis represent percentage. ^a^Statistically significant difference with respect to the control value and ^b^with respect to the value of Cd treated mice. *X* ^2^-test followed by Fisher's exact test; *P* < 0.05.

**Table 6:** Effect of *Arthrospira maxima *(AM) on the genotoxicity induced by cadmium (Cd) in pregnant mice and their fetuses.

Treatment (mg/kg)	Pregnant females		Fetuses	
	MNPE Mean ± SEM	MNNE Mean ± SEM	MNPE Mean ± SEM	MNNE Mean ± SEM
Water	1.66 ± 0.516^b^	29.33 ± 4.78^b^	5.16 ± 0.983^b^	31.83 ± 5.13^b^
Tween	1.83 ± 0.753^b^	28.83 ± 5.20^b^	4.33 ± 0.816^b^	28.00 ± 7.87^b^
AM (800)	2.16 ± 0.753^b^	34.00 ± 6.98^b^	4.66 ± 0.516^b^	29.33 ± 6.32^b^
Cd (1.5)	14.16 ± 1.472^a^	118.16 ± 11.33^b^	14.33 ± 1.366^a^	120.50 ± 17.54^a^
AM + Cd (200)	8.83 ± 1.169^a,b^	106.33 ± 16.02^a,b^	9.33 ± 1.033^a,b^	108.33 ± 15.31^a,b^
AM + Cd (400)	7.83 ± 0.753^a,b^	92.16 ± 10.24^a,b^	6.83 ± 0.753^a,b^	93.00 ± 10.79^a,b^
AM + Cd (800)	4.83 ± 0.408^a,b^	70.50 ± 14.43^a,b^	5.33 ± 0.816^a,b^	78.66 ± 9.61^a,b^

The determinations were made with 8 pregnant mice per group and in 4 fetuses per group. Micronucleated polychromatic erythrocytes (MNPE) were determined in 1000 polychromatic erythrocytes per mice, and micronucleated normochromatic erythrocytes (MNNE) were determined in 1000 normochromic erythrocytes per mice. Results are shown as the mean ± SEM. ^a^Statistically significant difference with respect to the control value and ^ b^with respect to the value of Cd treated mice. One-way ANOVA Student Newman Keuls tests; *P* < 0.05.
